# Multifunctional Lignin-Based Composite Materials for Emerging Applications

**DOI:** 10.3389/fbioe.2021.708976

**Published:** 2021-07-02

**Authors:** Chang Ma, Tae-Hee Kim, Kun Liu, Ming-Guo Ma, Sun-Eun Choi, Chuanling Si

**Affiliations:** ^1^Research Center of Biomass Clean Utilization, Engineering Research Center of Forestry Biomass Materials and Bioenergy, Beijing Key Laboratory of Lignocellulosic Chemistry, College of Materials Science and Technology, Beijing Forestry University, Beijing, China; ^2^Material Science and Engineering College, Northeast Forestry University, Harbin, China; ^3^Department of Forest Biomaterials Engineering, College of Forest and Environmental Sciences, Kangwon National University, Chuncheon-si, South Korea; ^4^Tianjin Key Laboratory of Pulp and Paper, Tianjin University of Science and Technology, Tianjin, China

**Keywords:** lignin, composites, carbon, synthesis, applications

## Abstract

Lignin exhibited numerous advantages such as plentiful functional groups, good biocompatibility, low toxicity, and high carbon content, which can be transformed into composites and carbon materials. Lignin-based materials are usually environmentally friendly and low cost, and are widely used in energy storage, environment, electronic devices, and other fields. In this review article, the pretreatment separation methods like hydrothermal process are illustrated briefly, and the properties and categories of technical lignin are introduced. Then, the latest progress of lignin-based composites and lignin-derived carbon materials is summarized. Finally, the current challenges and future developments were suggested based on our knowledge. It is expected that this review paper favored the applications of composites and lignin-derived carbon materials in the future.

## Introduction

**FIGURE 1:**
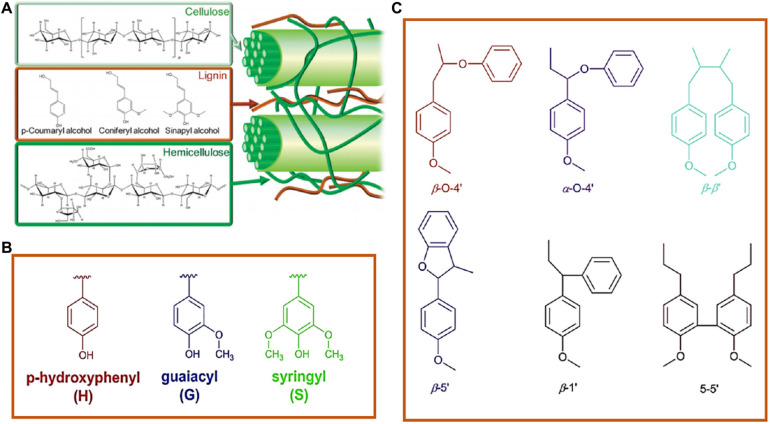
**(A)** Typical structure of lignocellulose (Zhu et al., 2020). **(B)** Precursors and **(C)** common interunit linkages (Luis Espinoza-Acosta et al., 2018).

Cellulose, lignin, and hemicellulose are the main chemical components of plant fiber raw materials (Li S.X. et al., 2019; Yang et al., 2019; Liu W. et al., 2020; Liu H. et al., 2021; Liu K. et al., 2021; Liu W. et al., 2021; Wang et al., 2021). Among these three components of lignocellulose, lignin is the only amorphous aromatic polymer (Figure 1A; Deuss et al., 2015; Xu J. et al., 2020; Ma et al., 2021b). Previously, the production of Kraft lignin and soda lignin was mainly used as a dye to provide a heat source for the burning section of the alkali recovery in the pulp and paper industry (Li et al., 2016; Xu et al., 2021). Given that lignin is a rich natural resource, increasing attention is paid to the research, development, and utilization of lignin in today’s increasingly scarce resources (Upton and Kasko, 2016; Li X. et al., 2019; Li et al., 2021). The structure of lignin is relatively complex than other biomass; therefore, it has a broad research prospect to develop appropriate methods for separation and refinery of lignin, conduct detailed research, and then unitize it to prepare materials rationally (Ragauskas et al., 2014; Chen et al., 2016; Meng L.Y. et al., 2019; Chen et al., 2020b; Ma et al., 2020).

Lignin is composed of three kinds of structural units such as syringl unit (S), guaiacol unit (G), and p-hydroxyphenyl unit (H) (Figure 1B; Decostanzi et al., 2019; Chen et al., 2020c; Shi and Ma, 2019; Liu K. et al., 2021). In the previous literatures, the lignin of softwoods is mainly G-type units; meanwhile, hardwoods are mainly G-type and S-type units. There are more abundant types of lignin in gramineous plants, including G, S, and H-type units. They are connected by ether bonds (about 60–70%) and carbon–carbon bonds (about 30–40%). Among them, all the alkyl-aryl ether bonds (β-*O*-4 and α-*O*-4), the β-β’ linkages, and the β-5 linkages are predominant between above three structural units (Figure 1C; Zheng et al., 2021). The structure composition and interunit linkages of lignin are also closely related to the external factors such as the growing environment of plants. Therefore, the different structural units, different linkage, and the complex relationship between lignin and glycan in the cell wall endow lignin one of the most complex natural polymers in nature. Lignin molecules contained a variety of active functional groups both on the benzene ring and the side chain, including aliphatic hydroxyl (Al-OH), phenolic hydroxyl (Ph-OH), carboxyl (-COOH), carbonyl (-C = O), and methoxy groups (-OCH_3_), determining the chemical properties and reactivity of lignin. The chemical properties of lignin allow it and its derivatives to be used as materials for value-high. Furthermore, considering the high carbon content of lignin, it is also an ideal carbon material precursor (Shi and Ma, 2019). Lignin-derived carbon materials are widely used in various fields like energy storage, adsorbent, and catalyst carriers (Suhas et al., 2007; Saha et al., 2014).

In this review article, we focus on the current achievements of lignin-based materials. The categories of lignin were introduced briefly. Then, the lignin-based materials like lignin-based hydrogels, flocculants, and resin adhesive, and lignin-plastic composites are summarized. In addition, the lignin-derived carbon materials such as activation carbon, carbon fibers, and carbon dots are discussed in detail. Finally, the existed problems and future trends of lignin-derived materials are proposed as well. It is expected that the lignin-based materials are promising applications in various fields.

## The Separation Methods and Compositions of Lignin

Lignocellulose is one of the most abundant biomass resources, mainly composed of 40–50% cellulose, 20–30% hemicellulose, and 25–35% lignin (Lievonen et al., 2016). According to statistics, about five thousand million tons of lignin has been produced globally every year (Chio et al., 2019; Meng Y. et al., 2019). Chemical structures of lignin varied among different plants species, such as softwoods, hardwoods, and grasses (Boerjan et al., 2003). Lignin does not stand for a single substance, but for a group of substance that have common properties in plants (Garcia Calvo-Flores and Dobado, 2010). The separation of lignin, based on the raw materials, can be divided into three types of separation from plant raw materials, separation from pulp, and separation from pulp waste liquid. Based on the separation principle, the first one is to remove the cellulose and hemicellulose by dissolution, leaving the insoluble residue of lignin. Meanwhile, the second is to dissolve the lignin, leave the insoluble residue of cellulose and hemicellulose, and recover lignin from the solution (Zhao and Abu-Omar, 2021). Bjorkman proposed a classic method for separating lignin by extraction after ball milling as early as 1953, resulting in the production of milled wood lignin. The milled wood lignin is closed to natural lignin, but in view of the yield is low, so it is often used to study the structure of lignin (Wang et al., 2009). Therefore, it is always a challenge to find a clean and efficient process to separate and recover lignin components with high yield and high structural integrity. Now, the research on biomass refining became a hot direction, which is to separate and extract lignin from biomass feedstock by pretreatment to make it easier for subsequent conversion and further applications. Numerous efforts have been devoted to find the potential pretreatment methods, and various methods have been explored, such as physical, chemical, physicochemical, and biological methods (Hochegger et al., 2019). For example, hydrothermal pretreatment is an environmental-friendly method for biomass separation. Sun et al. (2014) developed an integrated strategy including hydrothermal pretreatment and alkaline post-treatment, studied the changes of linkages during process, and obtained the highest yield of lignin up to 79.3%. These findings are beneficial to understand depolymerization and maximize the potential utilizations of lignin. In addition, there have been noticeable advances using novel solvents like ionic liquids, which are called “green solvents.” Since no toxic chemicals are formed and almost 100% can be recycled, it is considered that the ionic liquid pretreatment is a green solvent. Sun et al. (2019) applied a microwave-assisted ionic liquid approach to decrease the resistance of biomass in biorefinery and led to a high yield of lignin and efficient extraction of biomass. Deep eutectic solvent (DES) pretreatment is another new blooming green strategy for reducing biomass recalcitrance. Shen et al. (2019) employed biomass-derived DES including biomass-derived chemicals to deconstruct the structure of *Eucalyptus* for lignin valorization. Ma et al. (2021a) used microwave-assisted DES pretreatment to improve the lignin extractability and valorization of poplars. After DES pretreatment, the enzymatic saccharification rations were significantly increased, indicating that this microwave-assisted DES method could reduce the biomass recalcitrance and promote the lignin valorization. There have been series of review papers that summarize lignin extracted methods (Azadi et al., 2013; Chio et al., 2019). Herein, we mainly discuss the common industrial lignin.

In the paper industry, the four main methods of separating technical lignin (or pulping) are the Kraft pulping, sulfite pulping, soda pulping, and organosolv pulping processes. The obtained lignin types are Kraft lignin, lignosulfonate, soda lignin, and organosolv lignin, respectively (El Mansouri and Salvado, 2006). Due to the different processing methods, these four technical lignins have different structures, compositions, and properties. Kraft lignin is the residue of sulfate pulping in paper production, which is precipitated by adjusting the pH value of black liquor (Huang et al., 2017). The structure of Kraft lignin is highly modified and soluble in alkaline solution and organic solvents with high polarity (Chakar and Ragauskas, 2004). Lignosulfonate is sulfonated lignin, which is removed from the wood raw materials by sulfite pulping. Lignosulfonate is soluble in acidic solution, alkaline solution, and organic solvents with high polarity. Even though they contain sulfur, these two kinds of lignin have different characteristics, and the molecular weight of lignosulfonate is higher (Vishtal and Kraslawski, 2011). Soda lignin (or Alkaline lignin) is generally free of sulfur, which has a relatively lower molecular weight (Woermeyer et al., 2011). Organic solvents lignin is collected by organosolv pulping process, which has the characteristics of high purity, high homogeneity, and low molecular weight (Li and Takkellapati, 2018; Yu and Kim, 2020). However, the process includes the necessary solvent recovery steps, increasing the cost (Zhao and Abu-Omar, 2021).

## The Fabrication and Properties of Lignin-Based Materials

Owing to its good biocompatibility, ecological friendliness, and low toxicity, lignin is widely explored for high-value materials instead of burning (Si, 2019; Huang et al., 2020; Liu R. et al., 2020). The aromatic properties also make it possible to replace phenol to prepare phenolic resin adhesives (Pang et al., 2020; Pei et al., 2020). Herein, the synthesis and properties of lignin-based materials with various applications are described.

### Lignin-Based Hydrogels

Hydrogel is a kind of hydrophilic three-dimensional network gel, which can swell and hold large amounts of water. Forest biomass materials such as cellulose and hemicellulose are widely used in the preparation of hydrogels (Liu et al., 2017; Du et al., 2019; Li et al., 2020). Moreover, lignin is in its infancy as strength modifier, adhesive agents, or other functional fillers in hydrogels for lignin fractionation, wearable electronics, UV shielding, and biomaterials (Thakur and Thakur, 2015). Dai et al. (2019) fabricated a lignin-contained cellulose hydrogel for lignin fractionation. In this hydrogel, alkaline lignin was employed to play as a functional cross-linker to simultaneously improve the mechanical performances and realize specific absorbed or filtered. This lignin-cellulose hydrogel showed a reliable way to integrate lignin materials and lignin fractionation. Han et al. (2021) developed a polyvinyl alcohol (PVA) hydrogel with lignin-silver hybrid nanoparticles, which exhibited exceptional compressibility. As a strength modifier of hydrogel, lignin-silver hybrid nanoparticles provided strong hydrogen bonds and facilitated the electron transfer. Considering these outstanding traits of this PVA/lignin-silver hybrid nanoparticle hydrogel, this hydrogel could be used as a pressure-sensitive sensor to monitor signals. After demethylation, the phenolic hydroxyl groups of lignin have been released, which not only made the lignin with adhesion property but also improved the reducibility. Qian et al. (2021) took full advantage of this to reduce graphene oxide and develop a catechol lignin/reduced graphene oxide/sodium alginate/polyacrylamide double network hydrogel with integrated conductive, adhesive, and UV-blocking performance. The obtained hydrogel exhibited great potential in flexible electronic skin. Gao et al. (2021) designed a nanosilver immobilized glycine decorated lignin hydrogel as a catalyst, which showed outstanding catalytic performance of p-nitrophenol reduction. Amino modified lignin hydrogel networks played a role for catalyst carrier with abundant anchoring sites to disperse and stabilize the silver nanoparticles. After 10 cycles, the obtained catalyst can still maintain a catalytic efficiency of 98%, and there is no obvious collapse of the structure as well as the leaching of nanosilver can be ignored.

For the biomedical field, Zhang et al. (2020) assembled a biomimetic lignin/poly(ionic liquids) composite hydrogel by supramolecular interactions for the application of wound dressing. The resultant hydrogel exhibited satisfying mechanical strength, self-healing properties, bactericidal activity, and anti-oxidant activity. Lignin as reinforcement and antioxidant improved the mechanical enhancement and antioxidant activity of the hydrogel. Besides, lignin-based hydrogels have been used for the controlled release of drug (Witzler et al., 2018). Borisenkov et al. (2016) synthesized a hemicellulose and lignin composite hydrogel for drug delivery. Pectin was embedded in the hydrogel to form hydrophilic supramolecular complexes, which was employed to deliver β-glucuronidase and estrogens.

**FIGURE 2:**
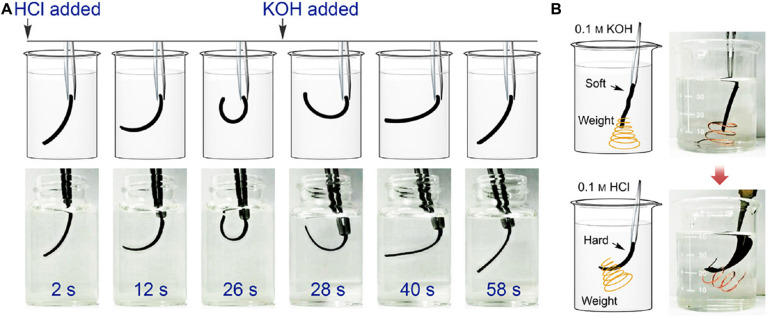
**(A)** pH-responsive deformation of the lignin-based hydrogel by adding HCl and KOH solution; **(B)** Actuating performance of the lignin-based hydrogel for being hooked up (Dai L. et al., 2020).

In addition, due to the changes of solubility, lignin can be used as pH-sensitive ingredient to form pH-responsive hydrogel in shape memory and controlled release (Figueiredo et al., 2017; Jin et al., 2018). Dai L. et al. (2020) prepared an all-lignin-based pH-stimuli-responsive hydrogel for the actuator. Herein, the kraft lignin was crosslinked with poly(ethylene glycol) diglycidyl ether to build this lignin hydrogel. As shown in Figure 2A, the lignin-based hydrogel bended spontaneously as the pH changes. Therefore, a mimetic behavior to hook up a wire has been achieved by adjusting pH (Figure 2B). These studies demonstrated that the use of lignin in hydrogel can contribute to areas such as electronics manufacturing, wearable devices, drug delivery, and actuators.

### Lignin–Phenol–Formaldehyde Resin Adhesive

From the perspective of the structural characteristics of lignin, it is also a high-value approach to prepare lignin–phenol–formaldehyde resin adhesive. Pang et al. (2017) studied the relationships between structure and property of two technical lignins in synthesis and performance of lignin–phenol–formaldehyde resin adhesive. They were obtained from acidic and alkaline organosolv pulping of bamboo. After purification, they were both characterized thoroughly, and the structural features were compared. The results showed that the long-chain hydrocarbon derivatives presented in lignin would affect the synthesis of lignin–phenol–formaldehyde resin.

**FIGURE 3:**
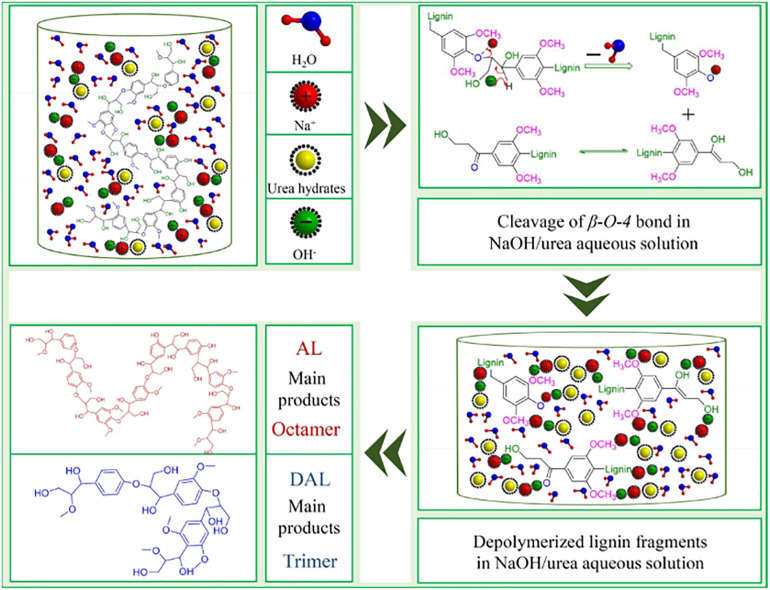
Schematic diagram of NaOH/urea aqueous solution to depolymerize the alkali lignin to prepare low molecular weight lignin derivatives (Li et al., 2018).

Depolymerization, activation, phenolate, and demethylation are the common pre-treatment processes to release the phenolic hydroxyl group of lignin (Naseem et al., 2016; Wang et al., 2018; An et al., 2019; Gan and Pan, 2019). For example, Ma et al. (2018) investigated a catalytic oxidative depolymerization process for increasing the content of phenolic hydroxy groups of Kraft lignin. Hydrogen peroxide and copper sulfate were used as catalysts in this process. After reaction, the phenolic hydroxyl content increased from 1.55 to 2.66 mmol g^–1^, and both the molecular weight and polydispersity decreased. The resultant lignin was used to synthesize lignin–phenol–formaldehyde resin with 50% substitution rate, whose various indexes all achieved the national standards. Base-catalyzed depolymerization of softwood Kraft lignin was used to release the phenolic hydroxyl of lignin to substitute phenol in resins (Solt et al., 2018). Modified renewable lignin-based phenols could replace phenol even at a high degree of substitution of 70%. As shown in Figure 3, Li et al. (2018) employed NaOH/urea aqueous solution to depolymerize the alkali lignin to prepare low molecular weight lignin derivatives, so as to further prepare lignin–phenol–formaldehyde resin. After depolymerization treatment process, phenyl-propane trimers were mainly obtained, and the phenolic hydroxyl group content increased from 0.07 to 0.12 mmol g^–1^. The resultant depolymerized alkali lignin–phenol–formaldehyde resin displayed fast curing rate, low formaldehyde emission, and high bonding strength. Microbes such as the brown-rot, white-rot, and soft-rot fungi were also investigated for the demethylation of Kraft lignin (Venkatesagowda and Dekker, 2020). Demethylation by the action of enzymes removed the *O*-methyl/methoxy of lignin and produced the demethylated Kraft lignin enriched in vicinal-hydroxyl groups, which has potential in lignin–phenol–formaldehyde resin. These studies demonstrated that the depolymerized lignin derivatives can replace phenol in the preparation of phenolic resin.

### Lignin-Based Flocculants

Lignin can be employed to treat wastewater. However, most of them suffered from poor solubility, chemical inactivity, and low molecular weight. Therefore, various chemical modification methods have been utilized to lignin to improve the flocculation performance (Wang et al., 2020b). Guo et al. (2018) developed an environmentally friendly lignin-based flocculant with improved flocculation by grafting the cationic acrylamide and dimethyl diallyl ammonium chloride monomers onto the alkaline lignin. The flocculation performance of the obtained lignin-based flocculant was low affected by pH. Moreover, the addition of Ca^2+^ and Mg^2+^ could significantly enhance the flocculation performance. Chen et al. (2020a) employed enzymatic hydrolysis lignin as raw materials, using polyacrylamide and methylacryloyloxyethyltrimethyl ammonium chloride as graft agent to synthesize a lignin-based cationic flocculant (L-CPA). The resultant L-CPA could self-assemble into octopus-like nanospheres, which endowed the high flocculation efficiency under the pH condition of 5–9. A small flocculant could be used to flocculate kaolin suspension. Such cheap, environmentally friendly, and technically feasible lignin-based flocculant exhibited a broad prospect in wastewater treatment process. Wang et al. (2020a) designed a lignin-based flocculant by mild copolymerization of lignosulfonate and [2-(methacryloyloxy) ethyl] trimethylammonium chloride solution. By changing the reaction conditions, two classes of flocculant were obtained, which were suitable for simulated dye wastewater (removal rate up to 95%), kaolin (turbidity removal rate up to 99.2%), and *Escherichia coli* suspensions (bacterial removal rate up to 97.5%), respectively. Anionic lignin-based flocculant was also prepared (Aldajani et al., 2021). Aldajani et al. (2021) prepared a hydrolyzed anionically modified lignin-acrylamide flocculant and investigated the different properties of polymer on the suspension’s attributes such as zeta potential, relative turbidity, flocs strength, and recoverability. Through the combination of many of its functional groups, namely, amide, carboxyl, and hydroxyl, it is observed that this lignin-based flocculant had a deeper adsorption on alumina particles than other polymers. These studies exhibited that the production and application of high-efficiency lignin-based flocculants are of great significance for resource conservation, low carbon footprint, and wastewater reuse.

### Lignin-Plastic Composites

**FIGURE 4:**
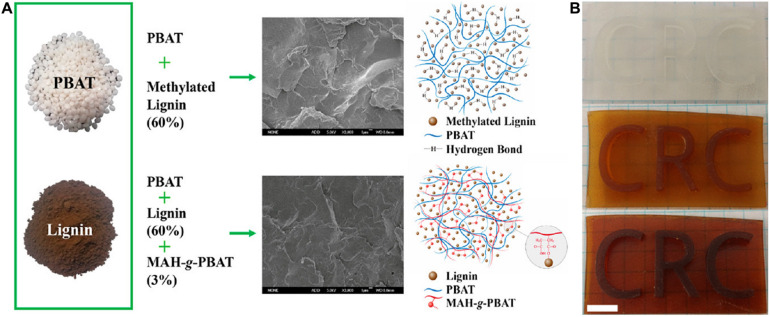
**(A)** Preparation of a composite by blending PBAT and Eucalypt hydrothermal lignin *via* two strategies (Xiong et al., 2020); **(B)** Digital photos of 0%, 5%, and 10% lignin (from top to bottom) exhibiting the effect on print quality (Sutton et al., 2018).

In past decades, billions of tons of non-biodegradable plastics have been produced, which is a significant source of pollution. As an abundant natural polymer, lignin could be integrated into plastics to fabricate high-value biodegradable materials with economic competitiveness (Sen et al., 2015; Kazzaz et al., 2019). Therefore, the preparation of composite materials by mixing lignin with various plastics had attracted attention. For example, Cerro et al. (2021) produced a poly(lactic acid) (PLA)/lignin nanoparticle composite containing cinnamaldehyde (Ci) for packaging and biomedical applications, which exhibited a better UV-light barrier property and biodegradable performance. Herein, lignin nanoparticles are used as fillers to enhance the mechanical strength of polymer composites. The toxicity of PLA/lignin composites has been studied as well, and the results showed normal blood parameters after a single dose of composites. Xiong et al. (2020) produced a composite by blending poly(butylene adipate-co-terephthalate) (PBAT) and Eucalypt hydrothermal lignin (Figure 4A). Two strategies were followed to improve the performance of composites, including methylated lignin replaced neat lignin as filler, and twin-screw extrusion was used as preparation method. The obtained PBAT/lignin composite materials exhibited a price advantage, in which the cost was significantly reduced by 36%.

Three-dimensional (3D) printing is a method of shape rendering. The ideal materials for 3D printing need to have good extrudability. The unique structures of lignin such as ether groups, β-*O*-4′ linkages, and oxygenated aromatic bonds endow it suitable to incorporate into conventional plastic materials to build hybrid materials by 3D printing with more environmentally friendly and better printability (Nguyen et al., 2018b). A study reported that organosolv hardwood lignin was mixed with nylon as 3D printing ink, and the lignin was found to improve the printability by reducing the melt viscosity and enhance the stiffness and tensile strength of the structure (Nguyen et al., 2018a). The proposed mechanism was lignin domains forming hydrogen bonds with the plastic matrix. This study came up with a new strategy of using biomass lignin as a feedstock for valuable 3D printing materials. Sutton et al. (2018) reported renewable, modified lignin-containing photopolymer resins for 3D printing by stereolithography. Compared to conventional photoactive resins, the lignin-containing resins displayed satisfied ductility, in which the lignin content can reach up to 15%. High print quality and visual clarity were obtained as shown in Figure 4B of the photographs of formulations with different lignin content. These studies showed that lignin is cheap and eco-friendly as a feedstock for plastic composites.

## Lignin-Derived Carbon Materials

Carbon materials were extensively applied in numerous fields such as energy storage and conversion, environmental applications, and catalyst (Dong et al., 2020). Generally, carbon materials are derived from petroleum-based chemicals by carbonization treatment, which is non-renewable, non-cyclable, and less environmentally friendly (Shi and Ma, 2019). Lignins are ideal raw materials as carbon precursors due to the low cost and high carbon content. It is of great significance to protect the environment, save resources, and develop the economy harmoniously.

### Lignin-Derived Activated Carbons

**FIGURE 5:**
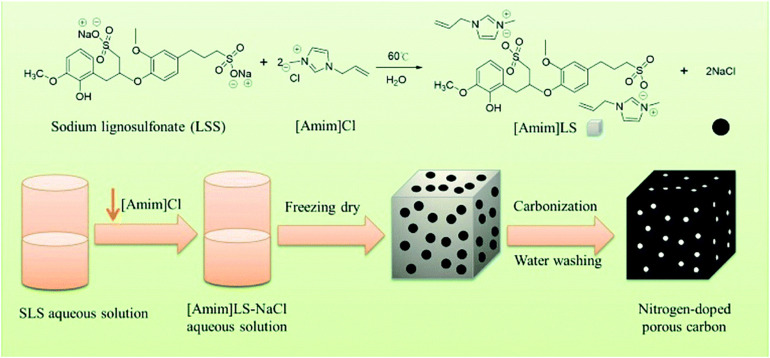
Schematic diagram of [Amim]LS precursors for N-doped porous carbon material fabrication (Xu Q. Q. et al., 2020).

Due to the high cost of producing activated carbon from coal, the production of activated carbon from lignocellulosic feedstock has attracted much attention (Jiao et al., 2021). A series of chemical activators like KOH and K_2_CO_3_ was adopted. For example, He et al. (2021) chose lignin-based pitch from black liquor as carbon precursor and KOH as chemical activator to synthesize porous activated carbon materials. The activation temperature on the lignin-derived active carbon was also explored. It was found that the maximum specific surface area and total pore volume reached the values of 3652 m^2^ g^–1^ and 2.35 cm^3^ g^–1^ under the activation temperature of 850°C. In addition, the ability of the lignin-derived activated carbon to absorb gaseous benzene has also been studied, and the adsorption performance exhibited that the carbon could be a good candidate for absorbing. As shown in Figure 5, Xu Q. Q. et al. (2020) employed sodium lignosulfonate (SLS) and ionic liquid ([Amim]Cl) to produce a new polymeric ionic liquid [Amim]LS and NaCl. The mixture was used as a precursor to prepare N-doped porous carbon material *via* direct carbonization without other activations. Herein, NaCl played the role of temple and activation agent. The obtained lignin-based porous carbon achieved a nitrogen content of 4.68%. Under the carbonization temperature of 700°C, a good energy density of 7.99 Wh kg^–1^ at the power density of 25 W kg^–1^ and cycling stability of 90.3% after 20000 cycles are shown. There are also some studies on the lignin-derived carbon with hierarchical porous architectures (Zhang et al., 2015a,b). Xi et al. (2021) obtained lignin-derived porous carbons with microstructural characteristics, high graphitization, high specific surface area, and hierarchical porosity for fabrication composites to alleviate the expansion and pulverization phenomena of lithium-ion batteries. Such lignin-derived porous carbons facilitated dispersing/coating of SnO_2_ and increased the reversible specific capacity from 64 to 620 mAh g^–1^. Wan et al. (2021) converted lignin to carbon materials with 3D hierarchical porous structures. After phosphoric acid plus hydrogen peroxide (PHP) oxidation pretreatment and KOH activation, the carbonized lignin reached a high surface area of 3094 m^2^ g^–1^ and pore volume of 1.72 cm^3^ g^–1^. The electrochemical measure results showed that the lignin-based carbon achieved a specific capacitance of 352.9 F g^–1^ at 0.5 A g^–1^, indicating an outstanding rate performance of this carbon electrode.

### Lignin-Derived Carbon Fibers

**FIGURE 6:**
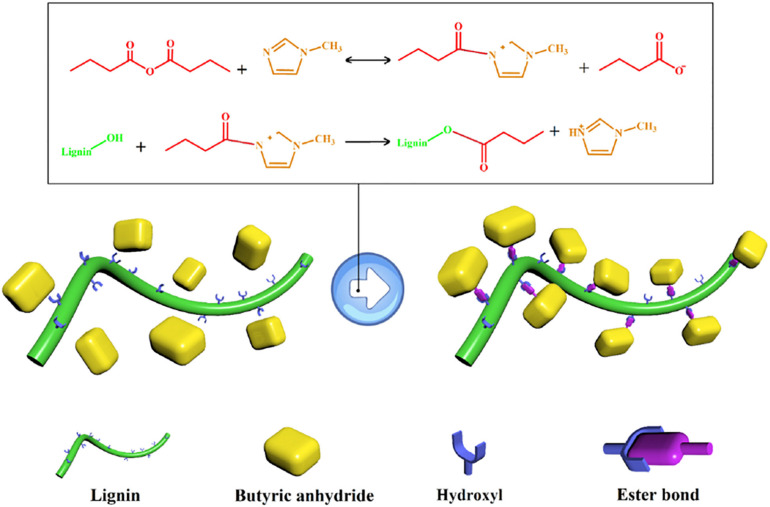
Illustration diagram of lignin esterification reaction (Dai Z. et al., 2020).

Lignin can be used as a cheap precursor in the preparation of carbon fibers instead of petroleum-based polymers by electrospinning technique and carbonization (Garcia-Mateos et al., 2019). Lignin-based carbon fibers with different functions can be obtained by adjusting the parameters of electrospinning, the template selected, and the materials loaded. For example, Ma et al. (2021c) prepared carbon nanofibers using lignin and polyvinylpyrrolidone as carbon precursor by electrospinning, peroxidation, carbonization, and pickling processes. Zinc nitrate hexahydrate was added and pyrolyzed to produce zinc oxide, which was used as a template to produce abundant micropores, resulting in the high specific surface area of 1363 m^2^ g^–1^. In view of the high specific surface area and abundant N/O groups, these lignin-derived carbon fibers with a specific capacitance of 289 F g^–1^ were seen as potential candidates for supercapacitor electrodes. Furthermore, the assembled symmetrical supercapacitor displayed outstanding cycling stability. Liu and Ma (2020) employed lignin as a renewable carbon source with polyacrylonitrile (PAN) and urea to prepare N-doped carbon nanofibers and then coated with polyaniline (PANI) for energy storage. The obtained lignin-based carbon fiber electrode displayed exceptional properties, including large specific surface areas of 483.1 m^2^ g^–1^, uniform pore size distribution of 9.1 nm, and specific capacitance up to 199.5 F g^–1^ at 1 A g^–1^. Eighty-two percent of the initial capacitance was maintained after 1000 charge/discharge at 4 A g^–1^. Dai Z. et al. (2020) developed a N, O co-doped carbon nanofibers (E-CNFs) from waste lignin and PAN by facile esterification and electrospinning method. The lignin esterification reaction was displayed in Figure 6, and the resultant esterified lignin had a low glass transition temperature for higher heteroatom content and better wettability of carbon nanofibers. E-CNF electrode exhibited a high capacitance of 320 F g^–1^ at a current density of 1 A g^–1^. An outstanding energy density of 17.92 Wh kg^–1^ at the power density of 800 W kg^–1^ was achieved by E-CNF symmetric supercapacitors.

In addition to energy storage, lignin-derived carbon fibers have been used in the field of catalysis as well. Lignin-based Pt supported carbon fiber electrocatalysts were prepared for alcohol electro-oxidation (Garcia-Mateos et al., 2017). Lignin/ethanol/phosphoric acid/platinum acetylacetonate solutions were chosen as precursors for electrospinning. After thermostabilization and carbonization at 900°C, carbon fibers with porous structure and Pt particle loading were obtained. Among them, the addition of phosphorus improved the oxidation resistance, avoided the oxidation of the lignin-based carbon fibers in the preparation process, and led to the generation of microporous architectures, which were beneficial to enhance the catalyst performance in the electro-oxidation of methanol and ethanol.

### Lignin-Derived Carbon Dots

Carbon dot is a novel type of carbon nanomaterial, which was found in 2004 (Xu et al., 2004; Kang et al., 2020). Zhang et al. (2019) prepared carbon quantum dots with bright green fluorescence by a simple one-pot route. Alkali lignin was employed as a precursor. Chao et al. (2021) employed lignin-derived carbon dots as photothermal thermogenesis materials to enhance wood-derived evaporation system. Herein, the lignin-derived carbon dots were obtained by hydrothermal method. An evaporation performance of 1.18 kg m^–2^ and efficiency up to 79.5% were achieved. Yang et al. (2020) developed a green approach to prepare sulfur-doped carbon dots by hydrothermal treatment of lignin. The obtained lignin-derived carbon dots possessed sulfur-containing groups, exhibiting good fluorescence with a quantum yield up to 13.5% and outstanding stability in acidic environments with a wide pH range of 0–5.0. Therefore, this lignin-derived carbon dots were successfully used in detection of Sudan I in acidic conditions.

## Conclusion and Perspectives

With the intensive investigation of lignin-based materials, the great development potential has been revealed in various fields. More and more efforts should be devoted on lignin-based materials and lignin-derived carbon materials. Further perspectives in lignin-based materials and lignin-derived carbon materials are proposed as follows.

(1) The low reactivity, solubility, and compatibility with conventional polymers of technical lignin enhance the difficulty of lignin to be a candidate to fabricate materials. Through chemical modification and careful design, these problems are partially or fully worked out, which expands the application of lignin in composite materials.
(2) Lignin does not stand for a single substance, but for a group of substances that have common properties in plants. Lignin is heterogeneous in nature, and it usually has heterogenous molecular weights, different functional groups, and different proportions of structural units. It is not conducive to repeatability, uniformity, and scalability of lignin-based materials. The obtained uniform lignin product *via* fractionation process may be one of the solutions for this problem.
(3) For lignin-derived activated carbon materials, chemical activators such as KOH and H_3_PO_4_ are often used to increase the specific surface area and the number of pores. However, most of these chemical activators are highly corrosive to the instrument and not recoverable. Therefore, it is vital to adopt green activators or design physical approaches for preparation of lignin-derived activated carbon.

(4) The morphologies of lignin-derived carbon materials are always disordered and uncontrollable. It is necessary to design hierarchical porous architectures according to different applications.
(5) For lignin composite materials, more advanced technologies and strategies should be developed, like 3D printing and screen process. In addition, other applications of lignin-derived materials should also be designed, such as nanogenerators, thermal management, biomedical field, and so on.

## Author Contributions

CM, KL, and M-GM: investigation. T-HK, S-EC, and CS: supervision. CM and M-GM: writing – original draft. M-GM, T-HK, KL, S-EC, and CS: writing – review and editing. All authors contributed to the article and approved the submitted version.

## Conflict of Interest

The authors declare that the research was conducted in the absence of any commercial or financial relationships that could be construed as a potential conflict of interest.
